# Studies of Dopamine Oxidation Process by Atmospheric Pressure Glow Discharge Mass Spectrometry

**DOI:** 10.3390/molecules28093844

**Published:** 2023-05-01

**Authors:** Dongli Dai, Yueqin Zhu, Zhenli Zhu, Rong Qian, Shangjun Zhuo, Anqi Liu, Xian Li, Wei Li, Qiao Chen

**Affiliations:** 1 National Center for Inorganic Mass Spectrometry in Shanghai, Shanghai Institute of Ceramics, Chinese Academy of Sciences, Shanghai 200050, China; ddongli5843@hotmail.com (D.D.); yqzhu@mail.sic.ac.cn (Y.Z.); sjzhuo@mail.sic.ac.cn (S.Z.); 1000511438@smail.shnu.edu.cn (A.L.); lixian1064@163.com (X.L.); 2 School of Material and Chemistry, University of Shanghai for Science and Technology, Shanghai 200093, China; liwei176@usst.edu.cn; 3 Center of Materials Science and Optoelectronics Engineering, University of Chinese Academy of Sciences, Beijing 100049, China; 4 State Key Laboratory of Biogeology and Environmental Geology, China University of Geosciences, Wuhan 430074, China; zlzhu@cug.edu.cn; 5 Department of Chemistry, School of Life Sciences, University of Sussex, Brighton BN1 9QJ, UK; qiao.chen@sussex.ac.uk

**Keywords:** dopamine, oxidation, atmospheric pressure glow discharge, characterisation, short-lived intermediates

## Abstract

An atmospheric pressure glow discharge ionisation source was constructed and utilized to study the dopamine (DA) oxidation process coupling with mass spectrometry. During the DA oxidation process catalysed by polyphenol oxidase (PPO), six cationic intermediates were directly detected by the atmospheric pressure glow discharge mass spectrometry (APGD-MS). Combined with tandem mass spectrometry, the structures of the dopamine *o-*semiquinone radical (DASQ) and leukodopaminochrome radical (LDAC^●^) intermediates and structures of the isomers of dopaminochrome (DAC) and 5,6-dihydroxyindole (DHI) were further characterised with the introduction of 2,2,6,6-tetramethylpiperidine 1-oxyl (TEMPO) and deuterium oxide (D_2_O) to APGD-MS. Meanwhile, UV–Vis studies confirmed the important role of PPO in catalyzing the DA oxidation reaction. Based on APGD-MS studies, a possible mechanism could be proposed for DA oxidation catalysed by PPO. Furthermore, APGD-MS could provide possibilities for the effective detection and characterisation of short-lived intermediates, even in complicated systems.

## 1. Introduction

Dopamine (DA) is a neurotransmitter that serves as a fundamental signalling chemical transmitter in intercellular communication [1,2]. Oxidation of DA is thought to be related to the aetiology of neurological diseases [3]. The detection of DA has become an important basis for the clinical diagnosis of many neurodegenerative diseases. Hence, it is critical to apply sensitive and accurate methods to characterise and understand the oxidative pathways and metabolites of DA. Many methods, including NMR [4], EPR [5], mass spectrometric [6,7] and spectroscopic techniques [8,9], have been applied to study the DA oxidation pathway. However, DA is highly active and easily undergoes autoxidation, metal oxidation, or enzymatic oxidation in the cytoplasm [10,11,12]. There are still many possibilities for the development of new techniques and applications for effective analysis of DA and its oxidation processes. 

Using mass spectrometry (MS) coupled with other techniques, the oxidation process of DA and the reaction mechanism were gradually understood and interpreted. In 2009, Zhou et al. improved tandem high-performance liquid chromatography coupled with electrospray ionisation MS to detect dopaminochrome (DAC) in the enzyme-catalysed dopamine process but did not isolate the isomers of DAC and 5,6-dihydroxyindole (DHI) [13]. In 2012, Liu et al. designed a method employing an interdigitated electrode coupled with nanospray desorption electrospray ionisation MS to detect the short-lived intermediate dopamine *o-*quinone (DAQ) of the electrooxidation of DA, capable of directly analysing small-volume electrolytes [14]. In 2017, Lftikthar et al. established a floating online electrochemical mass spectrometer using different solution compositions and MS detection conditions to identify three free radicals and the oxidation products of DA [15]. In 2020, Hu et al. combined bipolar ultramicroelectrodes (BUME) with nano-electrospray ionisation MS (nESI-MS) by fabricating a wireless carbon bipolar electrode into the tip of a nanopipette to identify the dopamine *o*-semiquinone (DASQ) [16]. A current report by Cao et al. developed an electrochemical-neutral reionisation MS (EC-NR-MS) technique for the study of DA oxidation, leading to the successful extraction and detection of the neutral *o*-semiquinone radical and the neutral leukodopaminochrome radical (LDAC^●^) [17]. Because of the possibility of matrix or salt effects in the buffer solution, the capture and characterisation of short-lived intermediates of DA oxidation was mostly performed by combination methods. Consequently, it is necessary to adopt new approaches for effective studies of the complicated intermediates of DA oxidation, especially under enzyme-catalysed conditions.

Ambient ionisation MS (AIMS) enables the direct and rapid surface analysis of short-lived species of different reactions [18,19]; one such approach that shows promise is atmospheric pressure glow discharge MS (APGD-MS) since it offers possibilities for direct and effective analysis of compounds without time-consuming pretreatment, auxiliary sampling, or desorption operations. Alves et al. reported an ion source for liquid sampling–atmospheric pressure glow discharge (LS-APGD) to produce ion intensities equal to or higher (>15%) than that of electrospray ionisation for marine-relevant salt-containing organic samples [20]. Fandino et al. evaluated a modified halo-shaped flowing atmospheric pressure afterglow (h-FAPA) source for direct analysis of benzene in volatile organic compounds with low matrix effects [21] and Zhang et al. designed a reactive-FAPA ionisation source for rapid isomer differentiation by real-time derivatisation of analytes [22]; moreover, Guć et al. analysed perfume components in cosmetics using FAPA-MS by directly determining the fragrance components: patchoulol, farnesol, safrole, geraniol, eucalyptol, and R-carvone [23]. These applications implied that the introduction of afterglow into APGD could not only extend the direct analysis of various types of samples [24], but also provide some possibilities for the identification of short-lived intermediates, even in complicated systems.

**Scheme 1:**
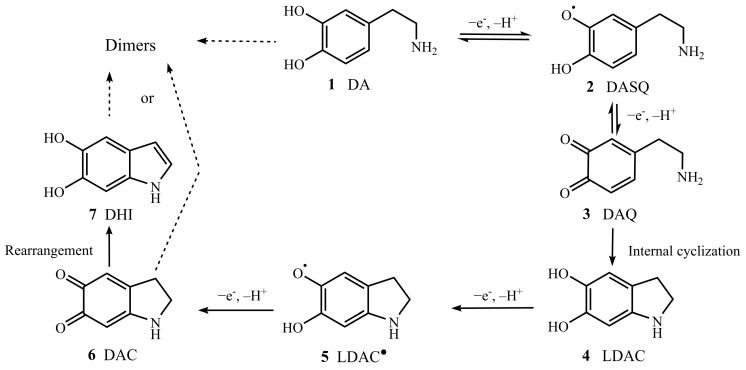
A possible mechanism for dopamine (DA)oxidation reaction catalysed by polyphenol oxidase (PPO). dopamine *o-*semiquinone radical (DASQ); dopamine *o-*quinone (DAQ); leukodopaminochrome (LDAC);leukodopaminochrome radical (LDAC^●^); dopaminochrome (DAC); 5,6-dihydroxyindole (DHI).

In the present study, APGD coupled with triple quadrupole MS was used to identify intermediates in the oxidation process of DA. To achieve high sensitivity for short-lived intermediates, the key parameters of APGD-MS were optimised at the beginning of the experiments and the DA species in solution were then directly detected during oxidation with polyphenol oxidase (PPO). The radical capture reagent 2,2,6,6-tetramethylpiperidine 1-oxyl (TEMPO) was used to verify the structure of two radicals, DASQ and LDAC^●^. In another experiment, deuterium oxide (D_2_O) was used in the reaction buffer in order to distinguish two isomers of DAC and DHI. Moreover, UV–visible spectrophotometer (UV–Vis) was also utilized to verify the catalytic ability of PPO. According to the previous literature, a possible mechanism for DA oxidation reaction catalysed by PPO was proposed in Scheme 1, which would be verified by the following studies.

## 2. Results and Discussion

### 2.1. APGD-MS Studies of DA Oxidation Process Catalysed by PPO

To obtain sufficient sensitivity and signal intensity of the short-lived intermediates, the operating parameters of the APGD-MS, including the gas flow rate (400 mL/min), interelectrode gap distance (6 mm), distance between the anode orifice and the MS inlet (14 mm), and discharge voltage (825 V), were previously optimised and fixed for the experiments. Under these conditions, APGD-MS was used to study the DA oxidation process catalysed by PPO [25]. The DA-PPO solution (2.5 mM DA mixed with 100 units (U) PPO in phosphate-buffered saline solution; PBS) was compared to a pristine DA solution (2.5 mM DA in deionised water).

**Figure 1:**
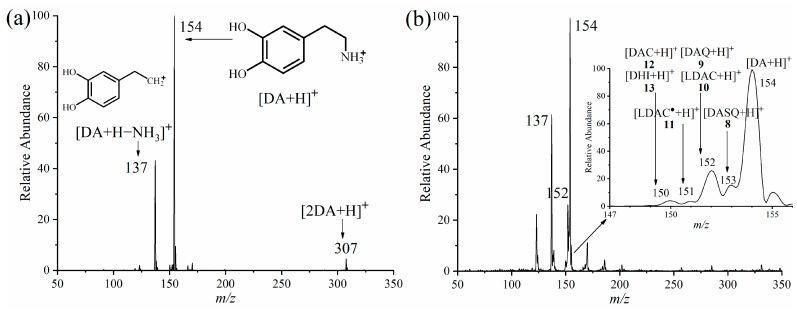
APGD-MS spectra of (**a**) DA pristine solution and (**b**) DA-PPO solution obtained in positive mode.

For the DA pristine solution, two ions assigned to [DA + H]^+^ at *m*/*z* 154 (principal ion of *m*/*z* 154.0861; calcd. for C_8_H_12_NO_2_^+^: *m*/*z* 154.0863; determined by ESI-Q-TOF-MS) and a fragment ion at *m*/*z* 137 from the protonated DA monomer were investigated by APGD-MS within 24 h, as shown in Figure 1a. Moreover, the peak at *m*/*z* 307 was assigned to the dimer of DA which was further characterised and confirmed by tandem MS Figure S2. For the DA-PPO solution, the oxidation of DA was initiated immediately after the PPO enzyme was added to the DA solution. Continuous monitoring of short-lived intermediates by APGD-MS allowed us to detect six cationic intermediates: the protonated DASQ radical cation **8** (principal ion of *m*/*z* 153, [DASQ + H]^+^, Figure 1b), the isomers of protonated DAQ and LDAC cations **9** and **10** (principal ion of *m*/*z* 152, [DAQ + H]^+^ and [LDAC + H]^+^, Figure 1b), the protonated LDAC radical cation **11** (principal ion of *m*/*z* 151, [LDAC^●^ + H]^+^, Figure 1b), and isomers of protonated DAC and DHI cations **12** and **13** (principal ion of *m*/*z* 150, [DAC + H]^+^ and [DHI + H]^+^, Figure 1b), (principal ion of *m*/*z* 150.0549; calcd. for C_8_H_8_NO_2_^+^: *m*/*z* 150.0550; determined by ESI-Q-TOF-MS).

**Figure 2:**
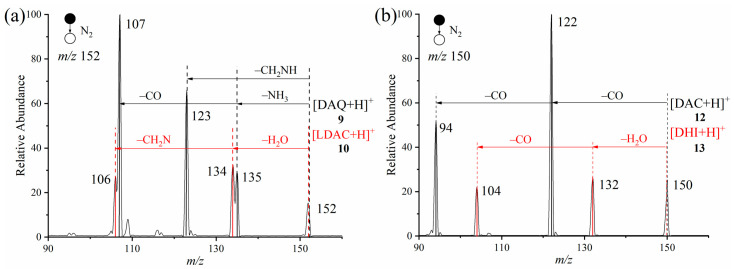
MS^2^ spectra of the ions (**a**) at *m*/*z* 152 and (**b**) *m*/*z* 150 in DA-PPO solution. The CID energy was 15 eV.

For further structural characterisation of the isomers of DA-PPO solution, ions with *m*/*z* 152 and *m*/*z* 150 were isolated for tandem mass spectrometric analysis (MS^2^). The MS^2^ experiment results suggested that the ions of *m*/*z* 152 produced five fragment ions at *m*/*z* 135, 134, 123, 107, and 106 by the loss of NH_3_, H_2_O, CH_2_NH, CO, and CH_2_N (Figure 2a), which was consistent with what the literature reports [14,15], and further demonstrated that the ions at *m*/*z* 152 could be assigned to the protonated isomers of intermediates **9** and **10**. Figure 2b suggests that the fragmentation pathway of one isomer of *m*/*z* 150 produced *m*/*z* 122 by loss of CO, which underwent ring fission and further generated *m*/*z* 94 by loss of CO [26]. The fragmentation pathway of another isomer of *m*/*z* 150 produced one fragment ion of *m*/*z* 132 by loss of H_2_O and one fragment ion of *m*/*z* 104 by loss of CO. The spectra were clearly different from the fragmentation pathway of **13** in the pristine DHI solution (Figure S1) and further demonstrated that the ions at *m*/*z* 150 could be assigned to the protonated isomers of intermediates **12** and **13**.

### 2.2. Verification of Protonated DASQ and LDAC Radical Cations with TEMPO by APGD-MS

**Figure 3:**
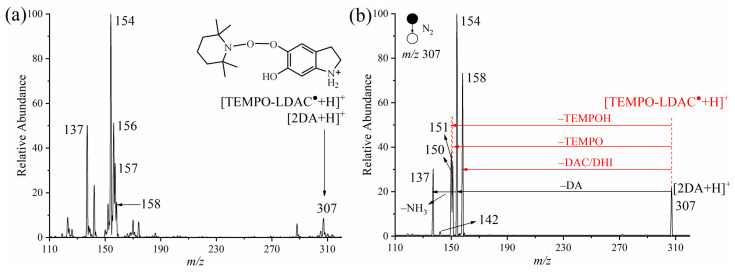
Spectra obtained from (**a**) APGD-MS of DA oxidation and (**b**) MS^2^ of the ion at *m*/*z* 307 in the DA-PPO solution with TEMPO. The CID energy was 2 eV.

To confirm the structures of the protonated DASQ and LDAC radical cations **8** and **9** (Figure 1b), TEMPO reagent was used to capture the radicals during the enzymatic oxidation of DA. In this experiment, 2.5 mM TEMPO was present in the solution containing 2.5 mM DA and 100 U of enzyme solution. Figure 3a shows the APGD-MS spectrum of the reaction: the peaks at *m*/*z* 156, *m*/*z* 157, and *m*/*z* 158 were assigned to the ammonium oxide ion of TEMPO, the oxoammonium salt, and the corresponding hydroxylamine (TEMPOH), respectively [27,28]. The peak at *m*/*z* 307 was assigned to a mixture of [TEMPO-LDAC^●^ + H]^+^ adduct and [2DA + H]^+^, which were further characterised by tandem mass spectrometry (Figure 3b).

According to the APGD-MS^2^ analysis results, the first possible fragmentation pathway of [TEMPO-LDAC^●^ + H]^+^ (principal ion of *m*/*z* 307.2016; calcd. for C_17_H_27_N_2_O_3_^+^: *m*/*z* 307.2016; determined by ESI-Q-TOF-MS) dissociated a DAC or DHI to produce [TEMPOH + H]^+^ at *m*/*z* 158, and the second possible fragmentation pathway of [TEMPO-LDAC^●^ +H]^+^ (*m*/*z* 307) dissociated a TEMPOH to produce [DAC + H]^+^ or [DHI + H]^+^ at *m*/*z* 150. Meanwhile, the possible fragmentation pathway of [2DA + H]^+^ was to dissociate DA to produce [DA + H]^+^ at *m*/*z* 154 and then dissociate NH_3_ to produce [DA + H-NH_3_]^+^ at *m*/*z* 137. Consequently, the MS^2^ experimental results confirmed the existence of [TEMPO-LDAC^●^ + H]^+^ as well as LDAC^●^ formed in the DA oxidation process. The TEMPO-LDAC^●^ adduct was detected by mass spectrometer for the first time and thus, MS^2^ was applied to identify its molecular structure. In addition, when the PPO enzyme was added, the peak intensity of the fragmentation ion at *m*/*z* 153 in Figure S3b was enhanced compared to that in Figure S3a in the MS^2^ pattern of *m*/*z* 309, which indicated the formation of intermediate cation **8** from DA oxidation. The peak at *m*/*z* 153 in Figure S3a might be due to intermediate cation **8** produced from the oxidation of DA by atmospheric oxygen [4]. After the addition of PPO enzyme, three new fragmentation ions (*m*/*z* 150, *m*/*z* 151, and *m*/*z* 158) were found in the MS^2^ pattern of *m*/*z* 307 (Figure 3b), indicating the formation of intermediate cation **11** from DA oxidation. Hence, the formation of these radical adducts was demonstrated, and protonated DASQ and LDAC radical cations **8** and **11** were determined by APGD-MS.

### 2.3. Verification of Isomers of Protonated DAC and DHI Cations with Deuterium Oxide by APGD-MS

**Figure 4:**
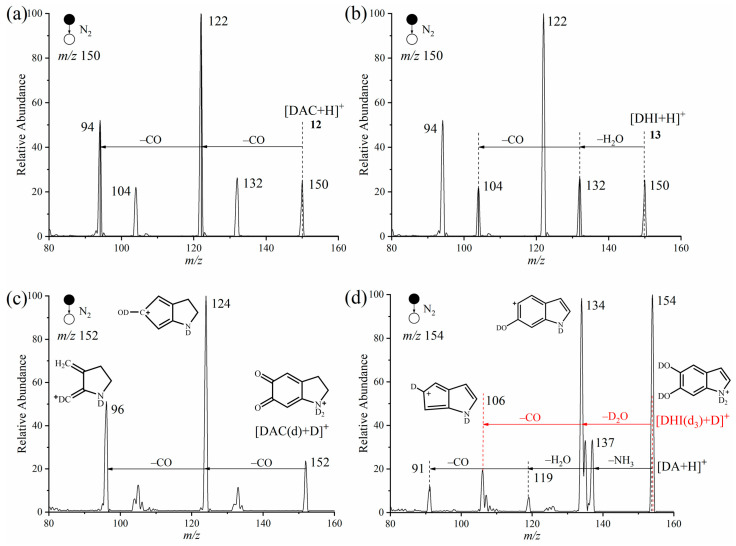
Spectra obtained from (**a**) APGD-MS of DA with D_2_O. MS^2^ spectra were obtained of (**b**) DAC and DHI in DA-enzyme solution; (**c**) DAC and (**d**) DHI in deuterated DA-enzyme solution. The CID energy was 15 eV, 15 eV, 20 eV and 15 eV for (**a**), (**b**), (**c**) and (**d**), respectively.

To further verify the structures of isomers of protonated DAC and DHI cations **12** and **13** (*m*/*z* 150) (Figure 1b), deuterium oxide (D_2_O) was introduced into a solution of DA and 100U PPO for MS^2^ experiments. Firstly, before adding the PPO solution, the [DA + H]^+^ can be deuterated into [DA (d_4_) + D]^+^ in the DA with D_2_O solution as shown in Figure S4. After the exchange of hydrogen and deuterium, the active H of DAC was changed by D, and the *m*/*z* changed from 150 to 152 of [DAC + D]^+^ (principal ion of *m*/*z* 152.0675; calcd. for C_8_H_6_D_2_NO_2_^+^: *m*/*z* 152.0667; determined by ESI-Q-TOF-MS). The MS^2^ experiments suggested that the possible fragmentation pathway of [DAC + D]^+^ was to dissociate CO to produce the first fragment ion at *m*/*z* 124 and continuously dissociate another CO to produce the second fragment ion at *m*/*z* 96 (Figure 4c) [29]. Meanwhile, the active H of DHI was replaced by D, and [DHI + H]^+^ at *m*/*z* 150 was changed to [DHI (d_3_)+ D]^+^ at *m*/*z* 154, which clearly separated DHI from DAC [30]. The following MS^2^ spectra suggested that the possible fragmentation pathway of [DHI (d_3_) + D]^+^ dissociates D_2_O to produce the first fragment ion at *m*/*z* 134 and continuously dissociates another CO to produce the second fragment ion at *m*/*z* 106. This is consistent with the MS^2^ spectrum of [DHI (d_3_) + D]^+^ at *m*/*z* 154 (Figure S5b). Another possible fragmentation pathway of [DA + H]^+^ produced three fragment ions with *m*/*z* 137, 119, and 91 by the loss of NH_3_, H_2_O, and CO, respectively. Consequently, protonated DAC and DHI cations **13** and **14** were also determined by APGD-MS. In this experiment, we introduced H/D exchange into APGD-MS to further improve the identification of enzyme-catalysed reaction intermediates.

### 2.4. Verification of PPO Activity during the DA Oxidation Process by UV–Vis Spectrophotometer

**Figure 5:**
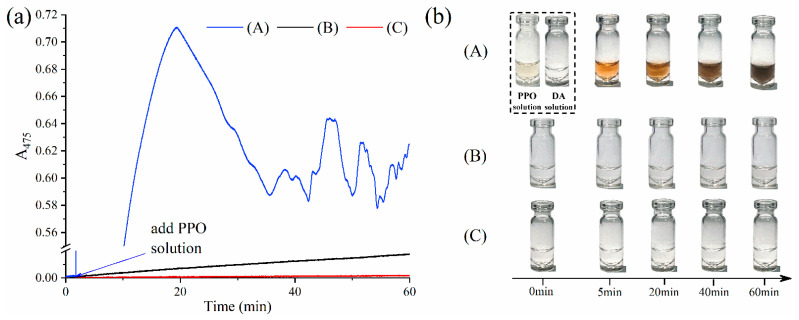
(**a**) A graph that is a curve of absorbance changes for a wavelength of 475 nm over time, (**b**) colour pictures of (**A**) DA-PPO solution, (**B**) DA-phosphate buffer solution and (**C**) DA pristine solution within 60 min.

To further verify the catalytic ability of PPO, the absorption wavelength of 475 nm assigned to DAC produced in the oxidation process of DA was detected by UV–visible spectrophotometer (UV–Vis) for three types of solutions. For DA-PPO solution, Figure 5a suggested that the absorbance of 475 nm increased sharply to maximum value of 0.7 after the addition of PPO, then decreased slightly to 0.6 and produced some fluctuations, which might be caused by the generation of melanin as the reaction proceeded [31]. Figure 5b indicated that the colour of reaction solution was pink at first and gradually deepened to be black around 20 min, which could confirm the generation of melanin for DA-PPO solution. Figure 5a also suggested that the variation value of absorbance was about 0.17 for DA-phosphate buffer solution and the absorbance of 475 nm changed very little for the DA pristine solution within 60 min. Meanwhile, UV–Vis studies could further verify that the PPO played a critical role in catalyzing the DA oxidation reaction, which was consistent with the results determined by APGD-MS.

### 2.5. Possible Mechanism of DA Oxidation Reaction Catalysed by PPO

**Scheme 2:**
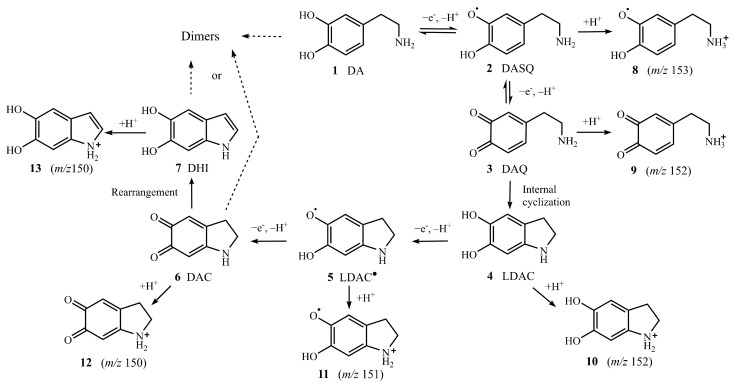
Proposed possible mechanism of PPO catalysed DA oxidation reaction.

Based on the above studies, six short-lived intermediate cations including **8**, **9**, **10**, **11**, **12** and **13** of the DA oxidation reaction catalysed by PPO were successfully characterised by APGD-MS method with TEMPO and deuterated reagents. Combined with other previous studies [32,33], a possible mechanism of DA oxidation reaction catalysed by PPO was proposed and shown in Scheme 2. Firstly, the DA released an electron and a proton to form intermediate 2, which might undergo disproportionation, one part was reduced to **1** while another part was oxidized to intermediate **3**. The intramolecular cyclization reaction of **3** was accompanied by the deprotonation of the amino group to form intermediate **4**. Transfer of one electron and one proton of **4** to generate intermediate **5**. Then, **5** was quickly oxidized to form intermediate **6**, which further rearranged to form **7**. Finally, the structural components were possibly bound together via covalent or noncovalent interactions to form a mixture of various oligomeric species with structural and redox disorder.

## 3. Materials and Methods

### 3.1. APGD-MS and UV–Vis Experiments

**Figure 6:**
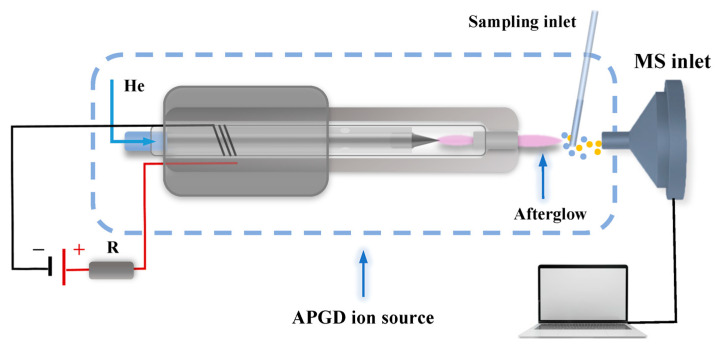
A schematic of the APGD-MS apparatus.

APGD-MS experiments were performed with a homemade ion source of APGD (Figure 6) coupled with a triple quadrupole mass spectrometer (Agilent Technologies, Santa Clara, CA, USA). The APGD ion source consisted of a quartz tube, titanium tube anode, and tungsten cathode. The APGD-MS experiments were carried out with the gas flow rate in the range of 100–600 mL/min, discharge voltage in the range of 765–1005 V, interelectrode gap distance in the range of 2–8 mm, and distance between the anode orifice and the MS inlet ranging from 3–15 mm. The basic parameters of MS were performed with *m*/*z* in the range of 10–500 and collision energy range in the range of 1–20 eV. The collision gas was nitrogen. The catalytic ability of PPO measured the absorbance at 475 nm by a UV–Vis spectrophotometer (SPECORD 50 PLUS, Analytik Jena, GA, Jena, Germany). The ESI-Q-TOF-MS (Agilent Technologies, Santa Clara, CA, USA) was determined to perform the experiments.

### 3.2. Materials and Reagents

Dopamine, PBS, and D_2_O were purchased from the Mackin Company (Shanghai, China). The compound 2,2,6,6-tetramethylpiperidine 1-oxyl was purchased from TCl (Shanghai) Chemical Industry Development Co., Ltd. (Shanghai, China). PPO (about 500 U/mg), from mushrooms, was purchased from Duly Biotech Company, Ltd. (Nanjing, China). Hirschmann^®^ minicaps capillaries (2 μL) conforming to ISO 7550, were obtained from Puyan Industrial (Shanghai) Co., Ltd. (Shanghai, China). Helium, with a purity of 99.999%, was used. A high DC power supply was obtained from Nantong Jiake Power Manufacturing Co., Ltd. (Nantong, China).

The PPO enzyme was dissolved in a 50 mM PBS (pH 7.4) to prepare enzyme solution with an activity of 1 U/μL. The reaction was initiated by adding 100 μL of PPO enzyme solution to 200 μL of a 2.5 mM DA solution in deionised water. For the analysis of free radicals, TEMPO (2.5 mM) was also utilized in the DA-PPO solution. The DA-PPO solution was also used for the isotopic labelling experiment, except the deionised water in the DA solution was replaced by D_2_O. All solutions were freshly prepared before the experiment began.

## 4. Conclusions

In conclusion, an APGD-MS method was developed for the efficient detection and characterisation of short-lived intermediates in PPO-catalysed systems. The intermediates, including **8**, **9**, **10**, **11**, **12**, **13**, appeared in Figure 1b at the same time in positive mode, and the MS^2^ spectra of **9**, **10**, **12**, **13** were obtained. The radicals, **8** and **11**, were successfully captured using TEMPO as a spin-trapping agent and were identified by APGD-MS. The isomers, **12** and **13**, were identified using deuterated reagents because of the mass difference of the active hydrogen. Meanwhile, UV–Vis studies could also verify that the PPO played a critical role in catalyzing the DA oxidation reaction, which was consistent with the results determined by APGD-MS. A possible mechanism of DA oxidation reaction catalysed by PPO was proposed based on APGD-MS studies with other previous studies. Thus, this represents a new strategy to characterise key intermediates with a short lifetime and generated during enzyme reactions. The APGD-MS strategy could also be used as a clinical diagnostic approach to detect the levels of dopamine and its oxidation products. Further studies in this area are currently being conducted in our laboratory.

## Data Availability

The data presented in this study are available upon request from the corresponding author.

## Supplementary Materials

The following supporting information can be downloaded from https://www.mdpi.com/article/10.3390/molecules28093844/s1, Figure S1: MS^2^ spectrum of the ion at *m*/*z* 150 in pristine DHI solution. The CID energy was 15 eV; Figure S2: MS^2^ spectrum of the ion at *m*/*z* 307 in DA/TEMPO solution. The CID energy was 2 eV; Figure S3: MS^2^ spectra of the ion at *m*/*z* 309 in (a) the DA/TEMPO solution and (b) the DA-PPO solution with TEMPO. The CID energy was 5 eV; Figure S4: APGD-MS spectrum in the DA with D_2_O solution before adding PPO or TEMPO; Figure S5: (a) APGD-MS spectrum and (b) MS^2^ spectrum of the ion at *m*/*z* 154 of deuterated DHI in the DHI solution. The CID energy was 15 eV.

## Sample Availability

Samples of the compounds are available from the authors.
