# Supplementary material for: Studies of Dopamine Oxidation Process by Atmospheric Pressure Glow Discharge Mass Spectrometry
Source: Molecules. 2023 May 1;28(9):3844. doi: 10.3390/molecules28093844 (PMC10179796; doi:10.3390/molecules28093844)
Supplement: Supplementary file 1 [file molecules-28-03844-s001.zip › molecules-2305145-supplementary.pdf]

Studies of Dopamine Oxidation Process by Atmospheric Pressure Glow Discharge Mass Spectrometry

Dongli Dai [1,2,3,†], Yueqin Zhu [1,3,†], Zhenli Zhu [4], Rong Qian [1,3,*], Shangjun Zhuo [1,3], Anqi Liu [1,3], Xian Li [1,3], Wei Li [2] and Qiao Chen [5]

[1] National Center for Inorganic Mass Spectrometry in Shanghai, Shanghai Institute of Ceramics, Chinese Academy of Sciences, Shanghai 200050, China; ddongli5843@hotmail.com (D.D.); yqzhu@mail.sic.ac.cn (Y.Z.); sjzhuo@mail.sic.ac.cn (S.Z.); 1000511438@smail.shnu.edu.cn (A.L.); lixian1064@163.com (X.L.)
[2] School of Material and Chemistry, University of Shanghai for Science and Technology, Shanghai 200093, China; liwei176@usst.edu.cn
[3] Center of Materials Science and Optoelectronics Engineering, University of Chinese Academy of Sciences, Beijing 100049, China
[4] State Key Laboratory of Biogeology and Environmental Geology, China University of Geosciences, Wuhan 430074, China; zlzhu@cug.edu.cn
[5] Department of Chemistry, School of Life Sciences, University of Sussex, Brighton BN1 9QJ, UK; qiao.chen@sussex.ac.uk
* Correspondence: qianrong@mail.sic.ac.cn; Tel.: +86-21-69163655
† These authors contributed equally to this work.

Supplementary Data

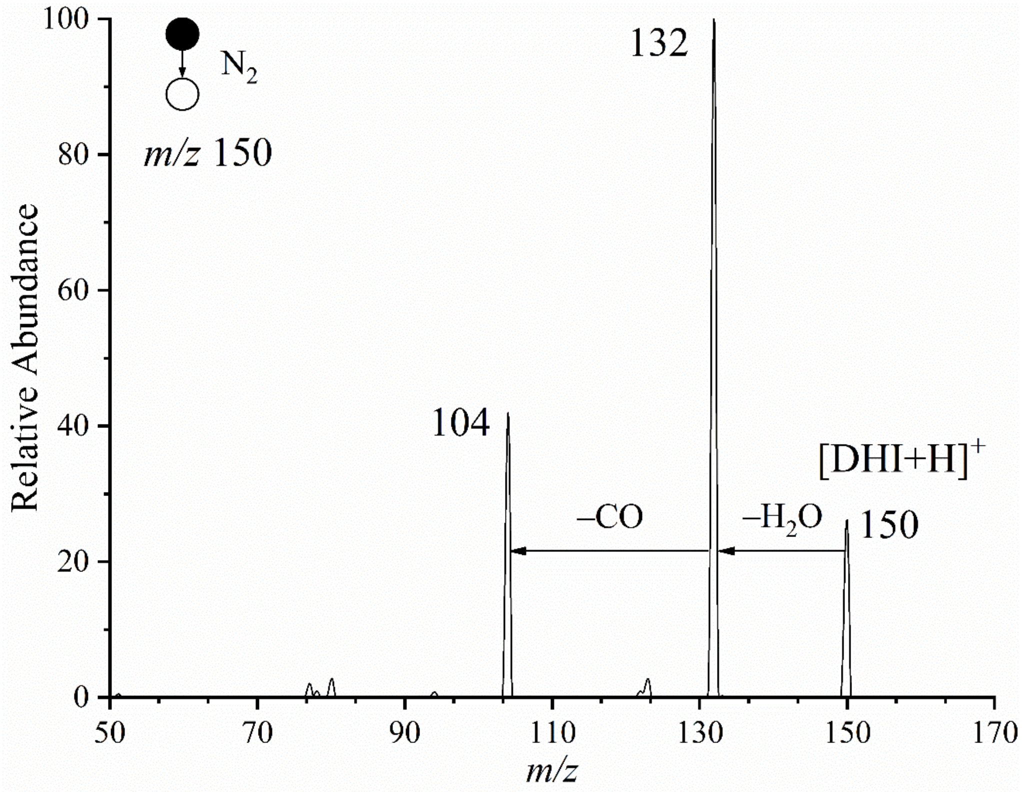

Figure S1. MS^2 spectrum of the ion at *m/z* 150 in pristine DHI solution. The CID energy was 15 eV.

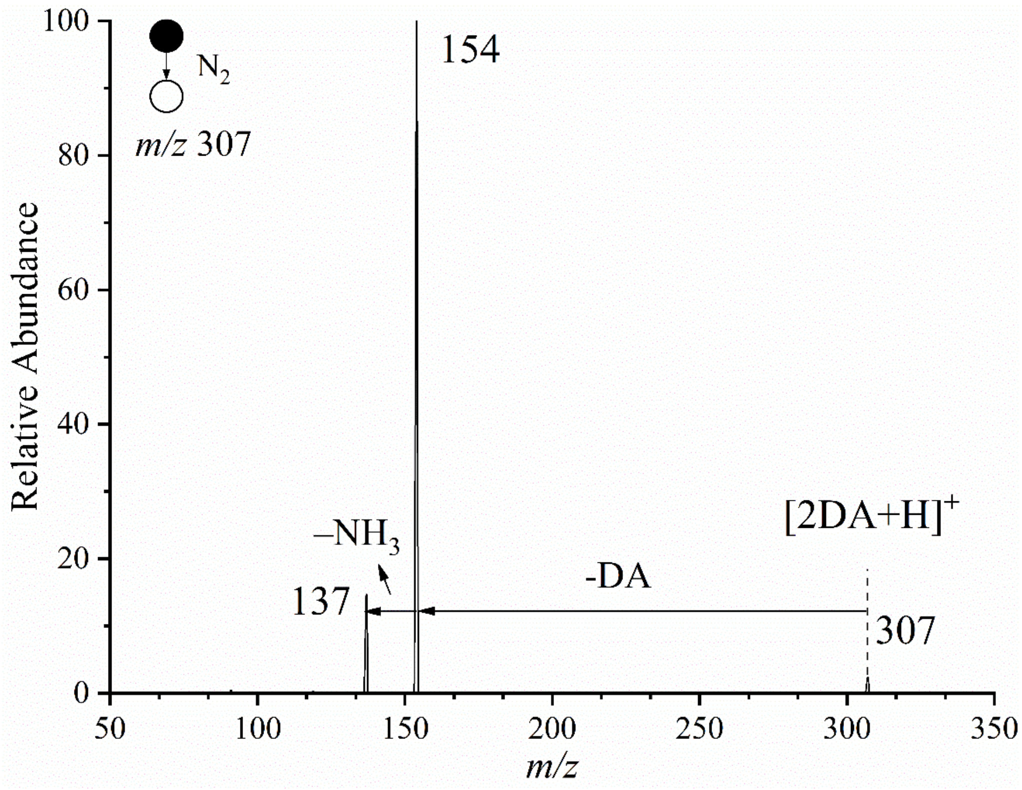

Figure S2. MS^2 spectrum of the ion at *m/z* 307 in DA/TEMPO solution. The CID energy was 2 eV.

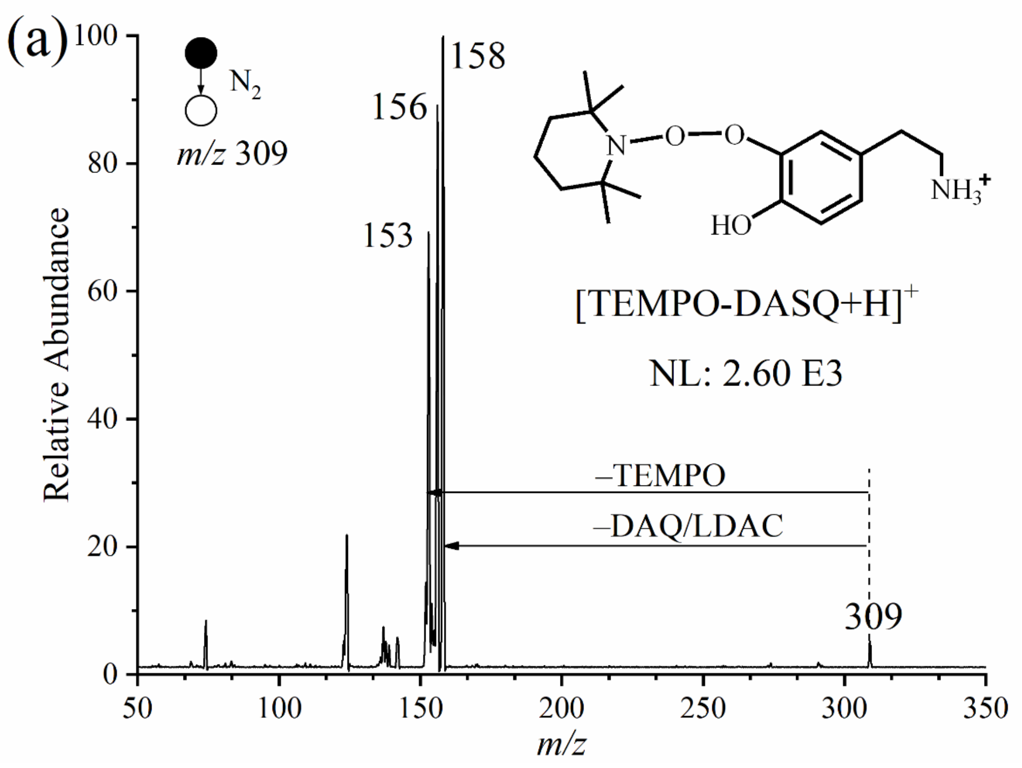

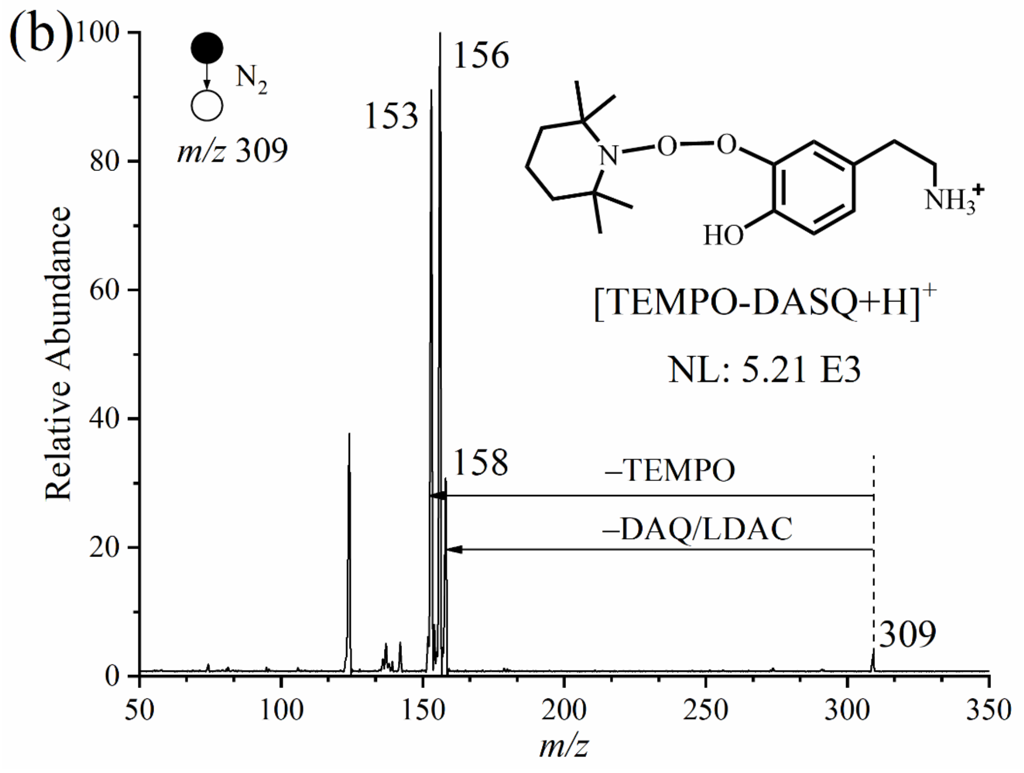

Figure S3. MS[2] spectra of the ion at *m/z* 309 in (a) the DA/TEMPO solution and (b) the DA-PPO solution with TEMPO. The CID energy was 5 eV.

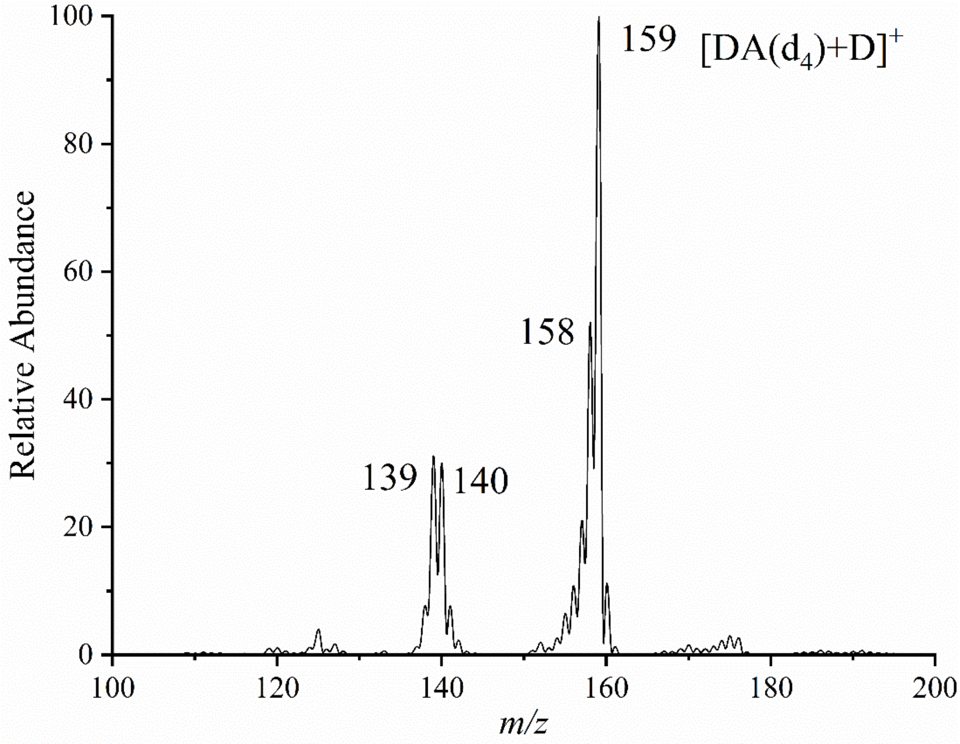

Figure S4. APGD-MS spectrum in the DA with D_2O solution before adding PPO or TEMPO.

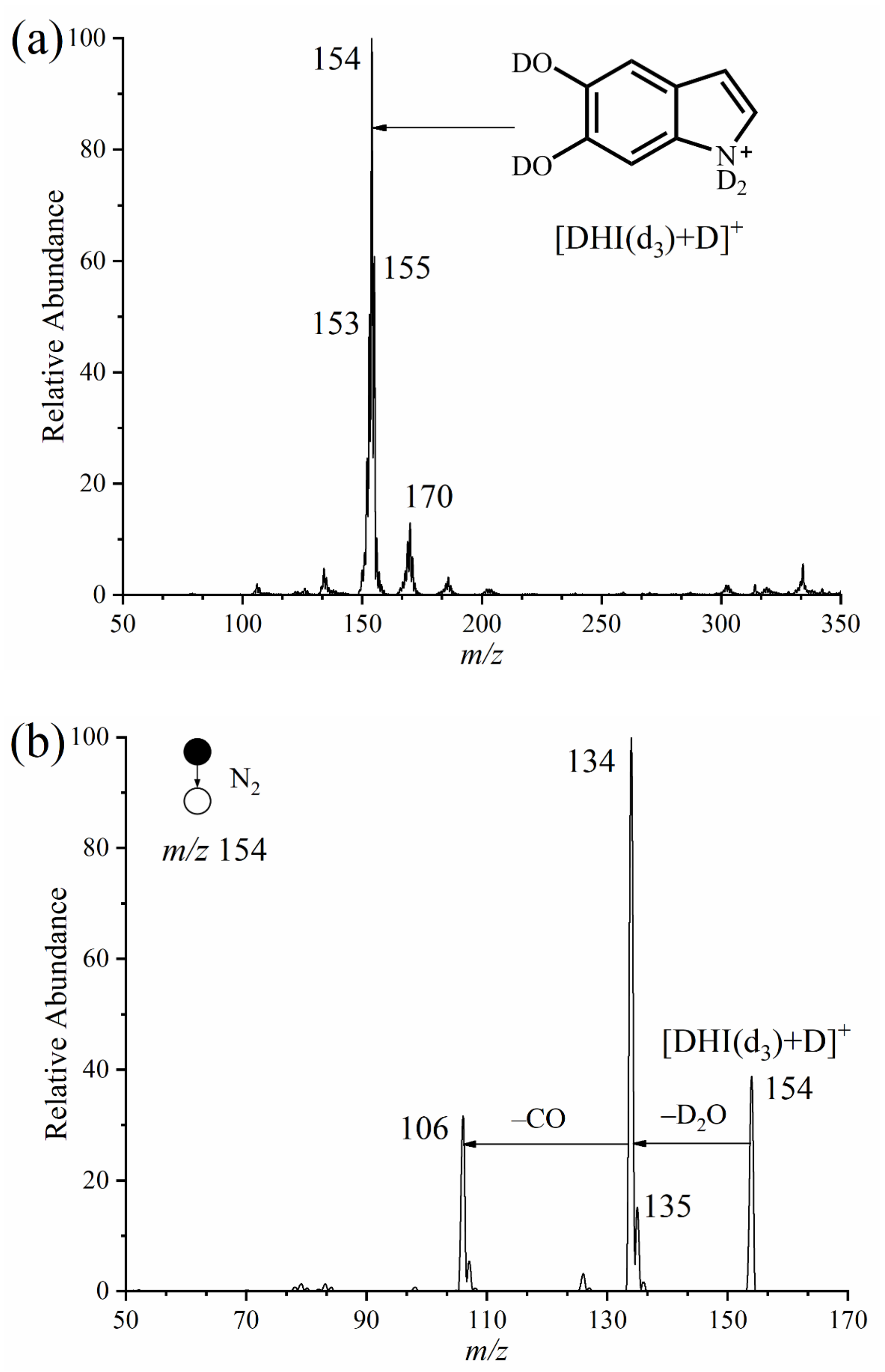

Figure S5. (a) APGD-MS spectrum and (b) MS^2 spectrum of the ion at *m/z* 154 of deuterated DHI in the DHI solution. The CID energy was 15 eV.
